# Trichostatin A enhances acetylation as well as protein stability of ERα through induction of p300 protein

**DOI:** 10.1186/bcr2562

**Published:** 2010-04-13

**Authors:** Sung-Hye Kim, Hyun-Jin Kang, Hyelin Na, Mi-Ock Lee

**Affiliations:** 1 College of Pharmacy, Seoul National University, San 56-1, Sillim-dong, Kwanak-gu, Seoul 151-742, Republic of Korea; 2 Bio-MAX Institute, Seoul National University, San 56-1, Sillim-dong, Kwanak-gu, Seoul 151-742, Republic of Korea; 3 Research Institute of Pharmaceutical Sciences, Seoul National University, San 56-1, Sillim-dong, Kwanak-gu, Seoul 151-742, Republic of Korea

## Abstract

**Introduction:**

Trichostatin A (TSA) is a well-characterized histone deacetylase (HDAC) inhibitor. TSA modifies the balance between HDAC and histone acetyltransferase activities that is important in chromatin remodeling and gene expression. Although several previous studies have demonstrated the role of TSA in regulation of estrogen receptor alpha (ERα), the precise mechanism by which TSA affects ERα activity remains unclear.

**Methods:**

Transient transfection was performed using the Welfect-EX™Plus procedure. The mRNA expression was determined using RT-PCR. Protein expression and interaction were determined by western blotting and immunoprecipitation. The transfection of siRNAs was performed using the Oligofectamine™ reagent procedure.

**Results:**

TSA treatment increased acetylation of ERα in a dose-dependent manner. The TSA-induced acetylation of ERα was accompanied by an increased stability of ERα protein. Interestingly, TSA also increased the acetylation and the stability of p300 protein. Overexpression of p300 induced acetylation and stability of ERα by blocking ubiquitination. Knockdown of p300 by RNA interference decreased acetylation as well as the protein level of ERα, indicating that p300 mediated the TSA-induced stabilization of ERα.

**Conclusions:**

We report that TSA enhanced acetylation as well as the stability of the ERα protein by modulating stability of p300. These results may provide the molecular basis for pharmacological functions of HDAC inhibitors in the treatment of human breast cancer.

## Introduction

Estrogen receptors (ERs) are members of a nuclear hormone receptor superfamily. ERs exist in two isoforms, ERα and ERβ, which have highly conserved DNA binding domains and ligand binding domains [1,2]. Although these receptors display similar binding affinities for 17β-estradiol, they have distinct roles in the regulation of gene expression and different interactions with unique sets of transcriptional factors [2]. Activation of ERα is considered a risk factor for the development of breast cancer, since the activation leads to cellular proliferation [3,4]. Cumulative data from tumor biopsies in the clinic have shown that two-thirds of breast cancers are ER-positive [5,6]. Tamoxifen, which regulates ERα activity, reduces the recurrence and death rate of ERα-positive breast cancer [7]. Breast cancer patients with expression of ERα are seven to eight times more likely to benefit from selective estrogen receptor modulators such as tamoxifen than ERα-negative patients [5]. ERα expression is therefore considered a significant outcome predictor for breast cancer patients to endocrine therapy.

The function of ERα is regulated by post-translational modifications such as phosphorylation [8,9], acetylation [10,11], sumoylation [12], and ubiquitination [13]. Among these modifications, acetylation is emerging as a central process in transcriptional activation of ERα [14]. ERα is directly acetylated by p300 at lysine 302 and 303 in the absence of ligand, and its acetylation regulates transcriptional activation and ligand sensitivity [10]. ERα is also acetylated at lysine 266 and 268 in the presence of coactivators p160 and p300, which enhances not only DNA binding but also transactivation activities. This acetylation was reversed by native cellular deacetylases, including trichostatin A (TSA)-sensitive class I and II histone deacetylases (HDACs), and nicotinamide adenine dinucleotide-dependent HDACs (class III, such as Sirt1) [11].

Generally, TSA is known to modify the balance between histone acetyltransferase and HDAC activities that induce histone hyperacetylation and regulate gene expression. Recently, the effect of TSA in acetylation/deacetylation of nonhistone proteins has been demonstrated as a diverse regulatory event, including ubiquitination/proteasomal degradation [15]. TSA effectively represses the mRNA and protein level of ERα in the ERα-positive breast cancer cells [16,17]. Although several previous studies have demonstrated the role of TSA-dependent HDACs in regulation ERα activity [18-20], the precise mechanism of TSA-induced activation of ERα remains unclear. We therefore explored whether TSA induces acetylation of ERα and increases stability of ERα in the present investigation.

## Materials and methods

### Cell and cell culture

The breast adenocarcinoma cell line T47D (ATCC HB 8065) and the human cervical carcinoma cell line HeLa (ATCC CCL-2) were obtained from the American Type Culture Collection (Manassas, VA, USA). Cells were maintained in Dulbecco's modified Eagle's medium containing 10% fetal bovine serum at 37°C in a 5% CO_2_/95% air incubator.

### Plasmids, siRNA and transient transfection

The Myc-tagged ERα, pCMV-Myc-ERα, was constructed by inserting a PCR-amplified full-length human ERα fragment into the *Eco*RI/Sall site of pCMV-Myc. The Myc-p300 expression vectors were gifted from Dr SC Bae (Chungbuk National University, Cheongju, Korea). The Myc epitope does not contain the known acetylated lysine residues [21,22]. Transient expression of proteins in HeLa cells was as described previously [23]. The siRNA duplexes targeting p300 and nonspecific siRNA (siGFP) were transfected as previously described [24,25].

### Western blotting and immunoprecipitation

Western blotting and immunoprecipitation were performed as previously described using specific antibodies against ERα, p300, Myc (Santa Cruz Biotechnology, Santa Cruz, CA, USA), and α-tubulin (Calbiochem, Darmstadt, Germany) [23]. To detect acetylated proteins, 500 μg whole cell lysates were incubated with 1 μg anti-pan-acetyl antibody (Santa Cruz Biotechnology) or anti-acetylated-lysine antibody (Cell Signaling Technology, Danvers, MA, USA), precipitated by adding 50 μl protein-A or protein-G agarose slurry, and then probed with specific antibodies or normal IgG. Acetylation of proteins was confirmed by reciprocal immunoprecipitation and western blotting. To detect ubiquitinated proteins, whole cell lysates were immunoprecipitated by 1 μg anti-ubiquitin antibody or anti-Myc antibody (Santa Cruz Biotechnology), and were probed using anti-Myc antibody or anti-ubiquitin antibody, respectively. Representative data from at least three independent experiments are shown.

### Reverse transcriptase-polymerase chain reaction

Total RNA was prepared using the Easy-Blue™ total RNA extraction kit (iNtRON Biotechnology, Seongnam, Korea) according to the manufacturer's instructions. PCR was performed as described previously using specific primers for ERα [26] and p300 [27]. The expression of β-actin was monitored as a control.

## Results

### Trichostatin A enhances acetylation as well as stability of ERα protein

Although previous studies have demonstrated that TSA-dependent HDACs regulated ERα activity, the precise mechanism remains unclear. We therefore explored the possibility that TSA induced acetylation of ERα and thereby affected the stability of ERα.

**Figure 1:**
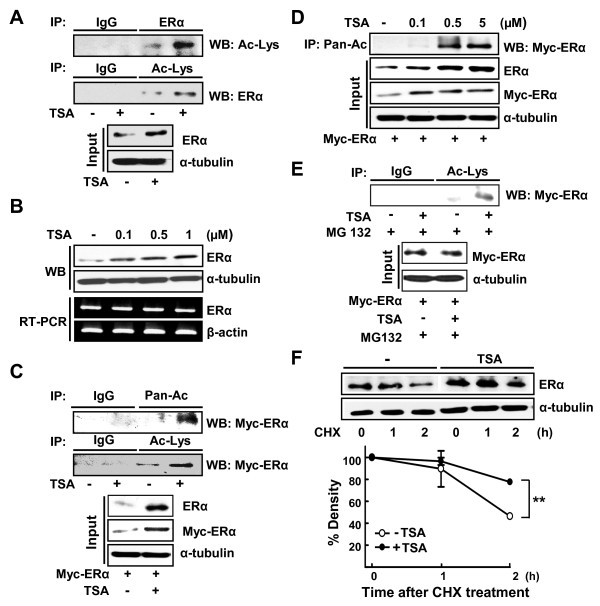
**Trichostatin A enhances acetylation as well as stability of ERα protein**. **(a) **T47D cells were treated with or without 1 μM trichostatin A (TSA) for 1 hour. Then 500 μg whole-cell lysate was immunoprecipitated (IP) using anti-ERα, anti-acetylated-lysine (Ac-Lys) or normal IgG antibodies, and western blot (WB) analyzed by anti-Ac-Lys or anti-ERα antibody as indicated. The expression of ERα and α-tubulin was analyzed by WB as input. **(b) **The ERα-positive T47D cells were treated with the indicated concentrations of TSA for 3 hours. The protein and mRNA expression of ERα were analyzed by WB and RT-PCR, respectively. **(c) **HeLa cells were transiently transfected with 4 μg Myc-ERα and then treated with or without 1 μM TSA for 3 hours. Then 500 μg whole-cell lysate was immunoprecipitated using anti-pan-Ac, anti-Ac-Lys or normal IgG antibodies, and probed by anti-Myc antibody. The expression of ERα was analyzed by WB using anti-Myc or anti-ERα antibodies as input. **(d) **HeLa cells were transiently transfected with 4 μg Myc-ERα and then treated with various concentrations of TSA for 3 hours. Then 500 μg whole-cell lysate was immunoprecipitated using anti-pan-Ac antibody, and probed by anti-Myc antibody. The expression of ERα was analyzed by WB using anti-Myc or anti-ERα antibodies as input. **(e) **HeLa cells were transiently transfected with 4 μg Myc-ERα and then treated with 1 μM TSA for 3 hours and 10 μM MG132 for 2 hours as indicated. Then 1 mg whole-cell lysate was immunoprecipitated using anti-Lys-Ac antibody, and probed by anti-Myc antibody. The expression of ERα was analyzed by WB using anti-Myc antibodies as input. **(f) **T47D cells were treated with or without 1 μM TSA for 3 hours in the presence of 10 μM cycloheximide (CHX) for the indicated time periods. At the end of treatment, whole-cell lysates were prepared and the expression of proteins was analyzed by WB. Density of the ERα protein band was determined using an image analysis system. The values were normalized to that of α-tubulin and expressed as a percentage of CHX-untreated control. Each data point represents the mean ± standard error of the mean of at least three independent experiments. **P < 0.01, -TSA vs. +TSA.

ERα was acetylated in the presence of 1 μM TSA when examined using anti-acetylated-lysine antibody in the human ERα-positive breast cell line, T47D (Figure 1a). We also found that the protein level of ERα was increased in the presence of TSA in a dose-dependent manner; the level was increased with as low as 0.1 μM TSA (Figure 1b). The mRNA level, however, was not altered. This result suggests that the TSA-induced ERα may be due to increases in protein stability, not increases in the transcriptional level.

We next studied the effect of TSA on the acetylation of ERα, expression of which is controlled by cytomegalovirus promoter using transient transfection of pCMV-ERα in HeLa cells. TSA also increased acetylation as well as the protein level of ERα in HeLa cells (Figure 1c, d). When acetylation was measured with similar amounts of ERα in the presence of an inhibitor of proteasome, MG132, the increase of acetylation in the presence of TSA was obvious, indicating that TSA affected protein stability but not transcription of ERα (Figure 1e). When the stability of ERα was measured using cycloheximide, a blocker of *de novo *protein synthesis, we observed that treatment of TSA blocked degradation of ERα (Figure 1f).

### Trichostatin A increases acetylation and protein stability of p300

It has been reported that ERα is directly acetylated by p300 at the well-conserved lysine residues in the hinge and ligand binding domain [10]. p300 acetylates itself, and the resulting conformational change modulates its transactivation potential [28,29].

**Figure 2:**
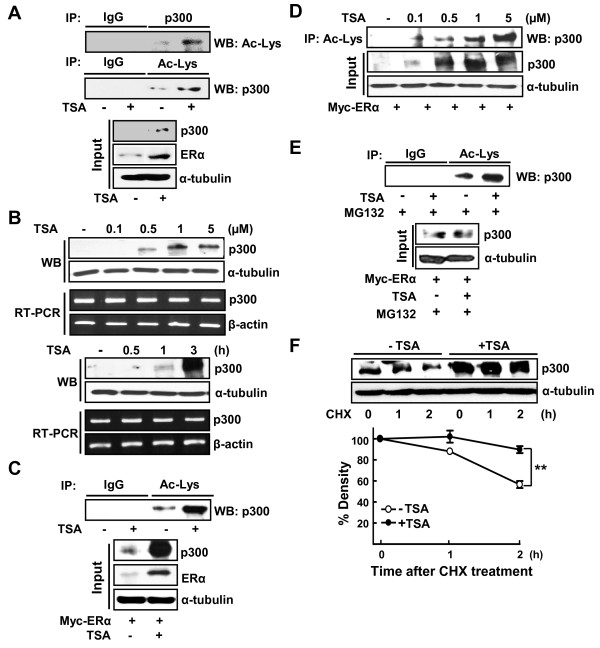
**Trichostatin A increases acetylation and protein stability of p300**. **(a) **T47D cells were treated with or without 1 μM trichostatin A (TSA) for 1 hour. Then 500 μg whole-cell lysate was immunoprecipitated (IP) using anti-p300 or anti-lysine antibody, and western blot (WB) analyzed by anti-p300 or anti-lysine antibody, respectively. The expression of p300 or anti-ERα was analyzed by WB using anti-p300 or anti-ERα antibodies as input. **(b) **T47D cells were treated with the indicated concentrations of TSA for 3 hours (upper) treated with 1 μM TSA for indicated period (lower). The protein and mRNA expression of p300 was analyzed by WB and RT-PCR, respectively. **(c) **HeLa cells transiently transfected with 4 μg Myc-ERα were treated with or without 1 μM TSA for 3 hours. Then 500 μg whole-cell lysate was immunoprecipitated using anti-acetylated-lysine (Ac-Lys) or normal IgG antibodies, and then probed by anti-p300 antibody. The expression of p300 and ERα was analyzed by WB using anti-p300 and anti-ERα antibodies as input. **(d) **HeLa cells were transiently transfected with 4 μg Myc-ERα and treated with various concentrations of TSA for 3 hours. Then 500 μg whole-cell lysate was immunoprecipitated using anti-Ac-Lys antibody, and probed by anti-p300 antibody. The expression of p300 was analyzed by WB using anti-p300 antibodies as input. **(e) **HeLa cells were transiently transfected with 4 μg Myc-ERα and then treated with 1 μM TSA for 3 hours and 10 μM MG132 for 2 hours as indicated. Then 1 mg whole-cell lysate was immunoprecipitated using anti-Ac-Lys antibody, and probed by p300 antibody. The expression of p300 was analyzed by WB as input. **(f) **T47D cells were treated with or without 1 μM TSA for 3 hours in the presence of 10 μM cycloheximide (CHX) for the indicated time periods. At the end of treatment, whole-cell lysates were prepared and the expression of proteins analyzed by WB. The density of p300 protein band was determined using an image analysis system. The values were normalized to that of α-tubulin and expressed as a percentage of CHX-untreated control. Each data point represents the mean ± standard error of the mean of at least three independent experiments. **P < 0.01, -TSA vs. +TSA.

We therefore investigated whether treatment of TSA increased the acetylation of p300 and thereby affected the acetylation level of ERα. As expected, acetylation of p300 was increased in the presence of TSA in T47D cells (Figure 2a). This observation suggests that the TSA-dependent HDACs are associated with deacetylation of p300. Similar to ERα, the protein level of p300 was significantly increased in the presence of TSA in a dose-dependent and time-dependent manner; however, the mRNA level of p300 was not altered (Figure 2b). Similar results were observed in p300 proteins overexpressed in HeLa cells (Figure 2c, d). The increase in acetylation of p300 was clearly observed in the presence of TSA when degradation of p300 was blocked by an inhibitor of proteasome, MG132 (Figure 2e). This result indicates that TSA affected protein stability rather than transcription of p300. The protein level of p300 was maintained by TSA in the presence of cycloheximide, indicating that TSA increases the protein stability of p300 (Figure 2f).

### p300 enhances the acetylation and stability of ERα protein

**Figure 3:**
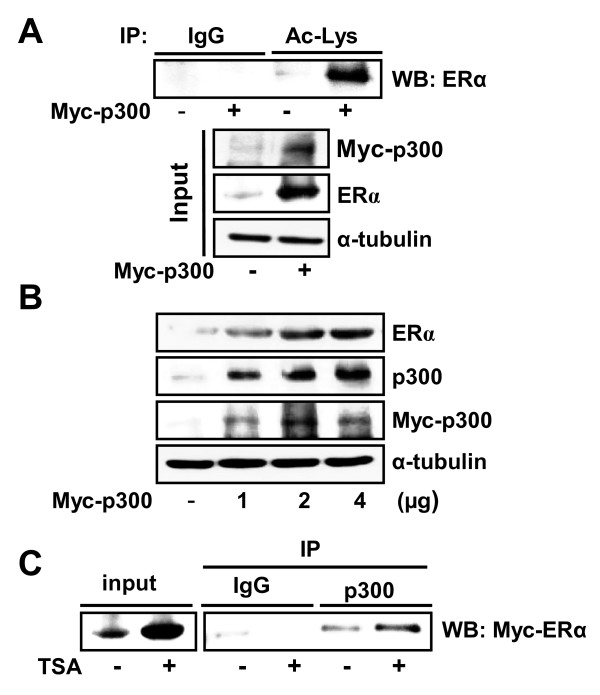
**p300 regulates acetylation and the protein level of ERα**. **(a) **HeLa cells were transiently transfected with 2 μg Myc-ERα with or without 2 μg Myc-p300. Then 500 μg whole-cell lysate was immunoprecipitated (IP) using anti-acetylated-lysine (Ac-Lys) or normal IgG antibodies, and probed by anti-ERα antibody. The expression of proteins was analyzed by western blot (WB) using anti-Myc or anti-ERα antibody as input. **(b) **HeLa cells were transiently transfected with 2 μg Myc-ERα or the indicated amounts of Myc-p300 or empty vector. The expression of proteins was analyzed by WB using anti-ERα, anti-Myc or anti-p300 antibodies. **(c) **HeLa cells were transiently transfected with 4 μg Myc-ERα and treated with or without 1 μM trichostatin A (TSA) for 3 hours. Then 500 μg whole-cell lysate was immunoprecipitated using anti-p300 or normal IgG antibodies, and probed by anti-Myc antibody.

We then examined whether p300 increased acetylation and stability of the ERα protein. When p300 was exogenously introduced together with ERα in HeLa cells, acetylation as well as the protein level of ERα were largely increased (Figure 3a). The increase in ERα depended on the amount of p300 expressed (Figure 3b). We also found that ERα was physically associated with p300 in the presence of TSA by co-immunoprecipitation assays (Figure 3c).

**Figure 4:**
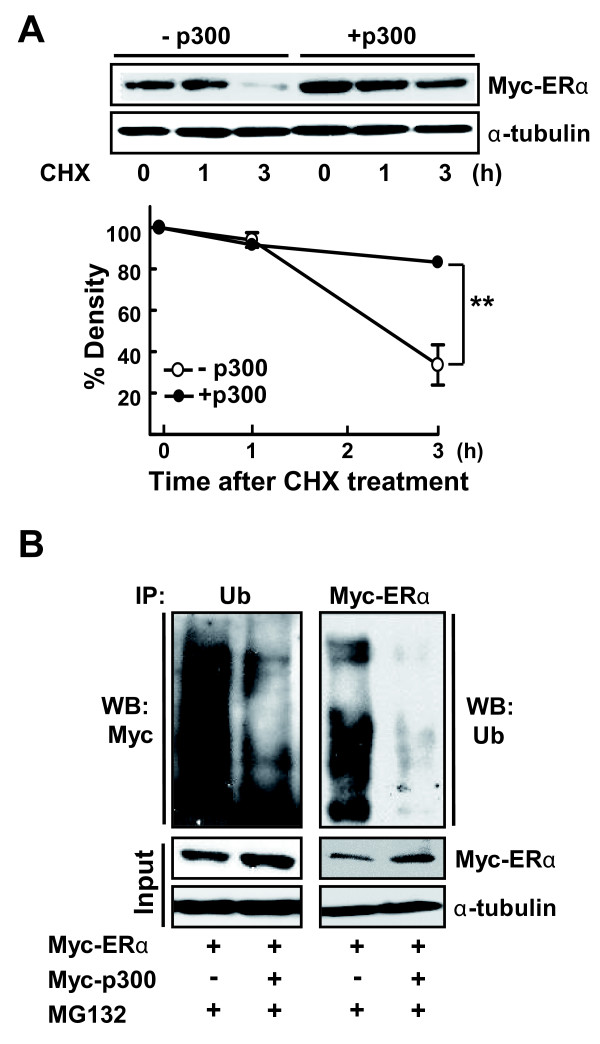
**p300 increases protein stability of ERα**. **(a) **HeLa cells were transiently transfected with 2 μg Myc-ERα together with 2 μg Myc-p300 or empty vector. At 24 hours after transfection, cells were treated with 20 μM cycloheximide (CHX) for the indicated time periods. The expression of protein was analyzed by western blot (WB) using anti-Myc antibody (upper). The density of ERα protein band was determined using an image analysis system. The values were normalized to that of α-tubulin and expressed as a percentage of CHX-untreated control (lower). Each data point represents the mean ± standard error of the mean of three independent experiments. **P < 0.01, -p300 vs. +p300. **(b) **HeLa cells were transiently transfected with 4 μg Myc-ERα together with 2 μg Myc-p300 or empty vector. At 24 hours after transfection, cells were treated with 10 μM MG132 for 2 hours. Then 500 μg whole-cell lysate was immunoprecipitated (IP) using anti-ubiquitin (Ub) or anti-Myc antibody and probed by anti-Myc or anti-Ub antibody, respectively. The expression of ERα was analyzed by WB using anti-Myc antibody as input.

The protein level of ERα was maintained in the presence of p300 while it largely decreased in the absence of p300, when cells were treated with cycloheximide for 3 hours (Figure 4a). Ubiquitination of ERα was dramatically repressed in the presence of p300. These results indicate that p300-induced acetylation of ERα is associated with blocking proteasomal degradation of ERα (Figure 4b).

**Figure 5:**
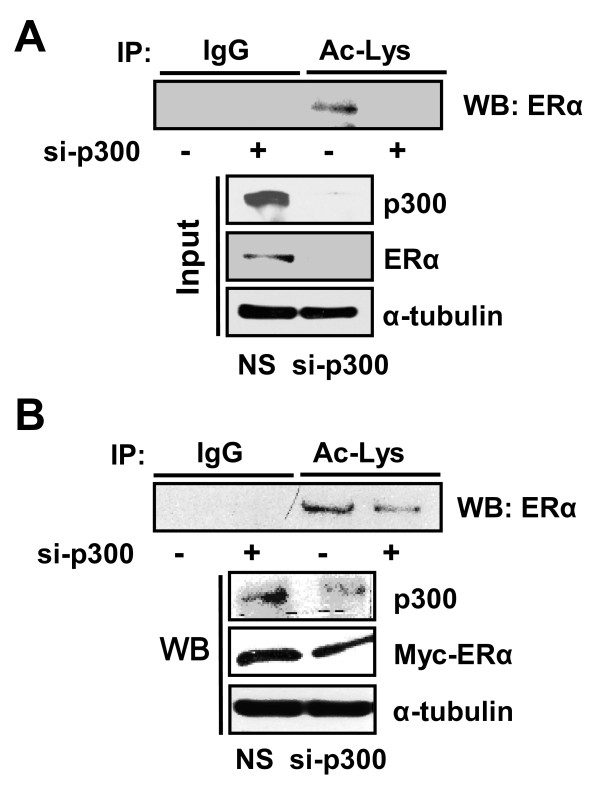
**Knockdown of p300 decreases acetylation and stability of ERα protein**. **(a) **T47D cells were transfected with si-p300 or nonspecific siRNA (NS) and treated with 1 μM trichostatin A (TSA) for 3 hours. **(b) **HeLa cells were transiently transfected with 2 μg Myc-ERα together with si-p300 or NS and treated with 1 μM TSA for 3 hours. Then 500 μg whole-cell lysate was immunoprecipitated (IP) using anti-acetylated-lysine (Ac-Lys) or normal IgG antibodies, and probed using anti-ERα antibody. The expression of p300 and ERα was analyzed by western blot (WB) using anti-p300 or anti-Myc as input.

Finally, we confirmed the role of p300 in TSA-induced ERα protein stability after knockdown of p300 using RNA interference. As shown in Figure 5, the TSA-induced acetylation and the increase in the protein level of ERα were largely diminished when expression of p300 was repressed, in both T47D and HeLa cells.

Together, these results demonstrated that TSA increased the stability of ERα protein by enhancing acetylation and stabilization of p300.

## Discussion

TSA not only inhibits growth of ERα-positive breast cancer cells *in vitro *but also inhibits breast tumor growth *in vivo *[16,17,30]. TSA may exert these beneficial effects against tumor growth by blocking deacetylation of histones and transcriptional factors, which subsequently alters transcriptional activity of target genes [15,31]. Here we report that TSA induces stability of ERα protein by enhancing acetylation and stability of p300, which may contribute to pharmacological effects of TSA.

Previous studies demonstrated that ERα is acetylated at multiple lysine residues, which may have different functions in the regulation of ERα activity. ERα was acetylated at lysine 266 and 268 in the presence of ligand in a steroid receptor coactivator-dependent manner [11]. ERα was also acetylated at lysine 302 and 303 in the presence of p300, and thereby regulated transcriptional activation and ligand sensitivity of ERα [10]. Ubiquitination at the same lysine residues was shown to regulate degradation of ERα [13]. The TSA-induced acetylation of ERα was accompanied with increased protein level of ERα (Figure 1), and p300 protected ubiquitination of ERα in our investigation (Figure 4b) - supporting the hypothesis that acetylation of ERα, probably at lysine 302 and 303 residues, is important for maintaining protein stability.

This observation is similar to the p300-induced acetylation of p53 or that of Smad7, which blocks ubiquitination and degradation of the protein [32,33]. Other nuclear receptors such as LXRα and AR are also present as acetylated forms that are involved in transactivation and other post-translational modifications such as ubiquitination [34,35]. Our finding contrasts, however, with previous reports that TSA downregulated the protein and mRNA level of ERα in ERα-positive breast cancer cells [16,17]. When MCF7 or T47D cells were treated with TSA for a prolonged period, we also observed a similar downregulation of ERα, indicating that TSA may affect ERα activity through at least two different mechanisms: transcriptional repression of the ERα promoter, and protein stability of ERα at the post-translational level. TSA may accomplish its beneficial effects against breast cancer by inducing sequential and/or divergent modifications of ERα at different regulation levels.

p300 was originally identified as E1A, which is a transcriptional co-activator for various transcription factors, including ERα [36]. The intrinsic histone acetyltransferase activity of p300 catalyzes acetylation of histone, which induces chromatin remodeling and subsequent transcriptional activation of target genes. p300 also acetylates nonhistone proteins such as p53 and Smad7, which leads to stabilization of target proteins [33,37]. Interestingly, the histone acetyltransferase domain of p300 acetylates itself [28]. Blanco-García and colleagues demonstrated recently that PCAF was acetylated by itself and by p300. Deacetylation of PCAF was catalyzed mainly by HDAC3, which affected subcellular localization of PCAF [38]. In the case of p300, acetylation was shown to increase transactivation activity and protein-protein interactions [28,29].

In the present study, we found another role for acetylation of p300 in the stabilization of p300 protein itself. Stabilization of p300 is induced within 3 hours of TSA treatment, which is similar to the TSA-induced acetylation and stabilization of ERα (Figure 2). Our finding, however, contradicts previous observations that autoacetylation of p300 did not alter its stability [29]. We believe this conflict may be due to different experimental conditions such as the expression level of p300 and the cell lines examined. Since p300 mediates acetylation of many proteins including histone and ERα, acetylation and subsequent stabilization of p300 may regulate the pharmacological effects of TSA through activation of diverse cellular transcription factors in breast cancer cells.

Our results together with those of other studies strongly suggest that the TSA-dependent HDACs are involved in acetylation of ERα [18-20]. The TSA-sensitive HDACs are class I and class II, which form a multiprotein repressor complex to remove the acetyl group from lysine residues of histones [39]. Indeed, HDAC1 and HDAC4, which belong to class I and class II, respectively, interacted with ERα and suppressed the transcriptional activity and expression of ERα [18,19]. On the contrary, nicotinamide adenine dinucleotide-dependent HDACs such as Sirt1 have been demonstrated to deacetylate ERα, probably at lysine 266 and 268, which enhanced the DNA binding and transactivation of ERα [11,40]. It would be interesting to know the unique function of each HDAC subtype as well as of each lysine residue in the regulation of ERα activity, such as protein stability and transactivation function. Especially, resveratrol - a Sirt1 activator - caused inhibition of estrogen-dependent cell proliferation, further supporting the notion that modifying ERα acetylation strongly influences epithelial cell growth in breast tissue [41]. Further molecular details of the acetylation of ERα and the resulting estrogen signaling could contribute to a novel therapeutic strategy against breast cancer.

## Conclusions

Several previous studies have demonstrated the role of TSA in regulation of ERα, but the precise mechanism of how TSA affects ERα activity remains unclear. We report that TSA enhanced acetylation as well as the stability of the ERα protein by modulating the stability of p300. Our results may provide the molecular basis concerning pharmacological functions of histone deacetylase inhibitors against human breast cancer.

## Abbreviations

ER: estrogen receptor; HDAC: histone deacetylase; PCR: polymerase chain reaction; RT: reverse transcriptase; TSA: trichostatin A.

## Competing interests

The authors declare that they have no competing interests.

## Authors' contributions

M-OL conceived of the study and its design, and interpreted the data. S-HK contributed to the study's conception and design, data collection and interpretation, and manuscript writing. H-JK and HN partly contributed to the study's design and data collection. Both authors read and approved the final manuscript.
